# Preconception Health Indicators and Deprivation: A Cross‐Sectional Study Using National Maternity Healthcare Data

**DOI:** 10.1111/1471-0528.18256

**Published:** 2025-06-19

**Authors:** Emma H. Cassinelli, Lisa Kent, Kelly‐Ann Eastwood, Danielle A. J. M. Schoenaker, Michelle C. McKinley, Laura McGowan

**Affiliations:** ^1^ Centre for Public Health School of Medicine, Dentistry and Biomedical Sciences, Queen's University Belfast Belfast UK; ^2^ University Hospitals Bristol NHS Foundation Trust Bristol UK; ^3^ School of Human Development and Health Faculty of Medicine, University of Southampton Southampton UK; ^4^ MRC Lifecourse Epidemiology Centre University of Southampton Southampton UK

**Keywords:** administrative data, inequalities, maternal health, population data, preconception health

## Abstract

**Objective:**

To use routinely‐collected maternity healthcare data to (1) describe the prevalence of key preconception indicators (e.g., smoking, folic acid supplement use) and (2) explore differences in prevalence by deprivation.

**Design:**

Retrospective population‐based study.

**Setting:**

Northern Ireland (NI).

**Population:**

255 177 pregnancies recorded in the Northern Ireland MATernity System (NIMATS).

**Methods:**

Anonymised NIMATS data recorded during antenatal booking appointments (2011–2021) were accessed through the Honest Broker Service and analysed using R. Prevalences were calculated for each indicator, and logistic regression models explored the relationships between each preconception indicator and area‐level deprivation quintiles. The indicators included were selected based on the current evidence base, availability in NIMATS, indicator modifiability and Patient and Public Involvement and Engagement.

**Main Outcome Measures:**

Preconception indicators, including behavioural factors (e.g., planned pregnancy), pre‐existing health conditions (e.g., severe mental health) and area‐based deprivation.

**Results:**

A high proportion of women had sub‐optimal preconception indicators (e.g., 21.3% living with obesity). Women living in the most deprived quintile generally had a higher prevalence of risk factors than women in the least deprived quintile (e.g., smoking prevalence was 25.7% in the most deprived quintile and 5.6% in the least deprived quintile).

**Conclusions:**

Population‐based maternity data in NI highlight many areas of women's preconception health that require improvement and support, especially for women living in the areas of greatest deprivation. Although these findings are a reference point to inform interventions, policy and ongoing monitoring of preconception health in NI, they should be interpreted in light of the methodological limitations of the data.

## 1 Introduction

Numerous indicators are known to characterise preconception health, which is the overall health of non‐pregnant individuals of childbearing age (15–49 years); these preconception indicators influence pregnancy and infant outcomes when conception occurs [1, 2, 3]. Indicators include health behaviours (e.g., folic acid supplement use), medical conditions (e.g., known pre‐existing physical and mental health conditions) and wider determinants of health (e.g., employment) [3]. The importance of these preconception indicators is gaining recognition among researchers, healthcare professionals (HCPs) and policymakers, both in the UK and globally. Concurrently, administrative data are increasingly being considered an important resource for healthcare research [4]. Using these data for research purposes can be advantageous owing to the large sample size available for analysis, population coverage and heterogeneity [5, 6, 7].

In 2019, the UK Preconception Partnership published a proposal for reporting key preconception health metrics, which serve to inform relevant agencies and governments on the delivery of preconception health‐promoting interventions [8]. This led to the publication of a ‘report card’ which presented the prevalence of various preconception indicators recorded in the national Maternity Services Dataset (England), including wider determinants of health, health behaviours, body mass index (BMI) and known family history [9]. Of all pregnant women attending their first antenatal appointment in England, around 90% were shown to have at least one indicator posing a risk to the mother and offspring, and individuals from lower socioeconomic levels were shown to have worse preconception health indicators overall [9]. Limited evidence is available on the prevalence of preconception indicators across the rest of the UK, and this is particularly important for Northern Ireland (NI), given it has the highest level of deprivation of all UK countries.

### 1.1 Research Aims

The current study aims to (1) describe the prevalence of prioritised preconception indicators recorded in the Northern Ireland MATernity System (NIMATS), described below, and (2) explore the relationship between area‐level deprivation quintiles and preconception indicators. These aims may facilitate the development of targeted preventative interventions to establish equitable preconception care and inform ongoing monitoring in NI.

## 2 Methods

A retrospective population‐based study was conducted using routinely‐collected maternity data from NIMATS. Ten indicators were prioritised, namely: area‐level deprivation (estimated using the Northern Ireland Multiple Deprivation Measure, NIMDM, 2017), planned pregnancy, smoking, folic acid supplement use, BMI derived by clinically measured weight and height at booking, alcohol consumption, diet quality, pre‐existing physical health conditions (e.g., hypertension), pre‐existing severe mental health conditions (e.g., schizophrenia) and previous obstetric complications (e.g., spontaneous miscarriage). These ten indicators were distilled from a longer list of 27 based on input from Patient and Public Involvement and Engagement (PPIE) representatives, as detailed below, recognising that the priorities of public members can differ from those of the scientific community [10, 11]. The initial list was informed by the availability of data in NIMATS and evidence‐based indicators from the literature [1, 9, 12, 13].

### 2.1 Data Source

The NIMATS dataset is a population‐level data repository containing maternal demographic and healthcare information for pregnancies conceived by women cared for within maternity services in NI [14]. It is available in all five Trust areas in NI.

Following data access approval by the Honest Broker Service (HBS), anonymised individual‐level data were accessed remotely via the UK Secure e‐Research Platform by accredited researchers (E.H.C., L.M., L.K.) Ethical approval and participant consent were not required.

### 2.2 Details of Indicators

This study included data from women who had a first antenatal appointment (and, when available, a recorded pregnancy outcome) between January 2011 and December 2021. Numerous preconception indicators were self‐reported to a HCPs during the initial booking appointment (e.g., pregnancy planning) or transcribed by HCPs (e.g., midwives transferring information from General Practice referral letters and healthcare records). Certain data were retrospective (e.g., previous obstetric complications), whereas other data represented women's characteristics at their booking appointment (e.g., BMI). Although these were collected at the early stages of pregnancy (the first antenatal appointment typically occurs between 10 and 12 weeks gestation) [15, 16], they are relevant for the characterisation of women's preconception health as many health behaviours and risk factors associated with adverse pregnancy outcomes are established during the period preceding pregnancy [3]. A midwife consultant familiar with data collection processes in healthcare settings was contacted to provide further context on the data, where applicable.

The data quality varied across the indicators used; while some allowed for more robust conclusions due to objectively measured data (e.g., BMI derived from height and weight measured at booking appointments), others should be interpreted with caution as they lacked objective or validated measurement instruments (e.g., diet quality). Details of the 10 indicators reported in this study are presented in Table 1 (further details in Supporting Information S1). Given this study's focus on health prior to conception, pregnancy and infant outcomes are not reported.

**TABLE 1:** Details of 10 priority indicators.

Indicator	How is it recorded in NIMATS?	How is it presented in this study?
Deprivation quintile	Area‐level deprivation was estimated using the Northern Ireland Multiple Deprivation Measures (NIMDM) 2017, which aggregates the ranking of seven specific domains into a single ranking. These weighted domains are: 25% income, 25% employment, 15% health and disability, 15% education, skills and training, 10% access to services, 5% living environment and 5% crime and disorder [17]. Maternal data in NIMATS were linked to the NIMDM by the super output area code [18]	Area‐level deprivation was categorised into quintiles, where the first quintile represented the most deprivation and the fifth the least deprivation
Planned pregnancy	Planned pregnancies were recorded as a binary yes/no measure at booking.	Planned pregnancies were presented as a yes/no measure
Smoking	Smoking status was calculated based on the reported number of cigarettes smoked per day at booking	Smoking status was presented as a yes/no measure
Folic acid supplement use	Folic acid supplement use was categorised based on both the timing (i.e., preconception and postconception) and dose reported (i.e., 400 μg or 5 mg). The data included for this measure were only pertaining to pregnancies with a booking appointment date after 01/12/2014, because of changes in the recording in NIMATS	Folic acid supplement use was presented as preconception 400 μg, preconception 5 mg, postconception 400 μg, postconception 5 mg and none
BMI	BMI was derived by clinically measured weight and height recorded at booking	Women were divided into: underweight (< 18.49 kg/m^2^), healthy weight (18.50–24.99 kg/m^2^), overweight (25.00–29.99 kg/m^2^), with obesity class I (30.00–34.99 kg/m^2^), with obesity class II (35.00–39.99 kg/m^2^) and with obesity class III (≥ 40.00 kg/m^2^) [19]. Recordings of BMI were included if in the range of 14–70 kg/m^2^
Alcohol consumption	Alcohol consumption was calculated based on the reported units of alcohol consumed per week at booking	Alcohol consumption was presented as a yes/no measure
Diet quality	Diet quality was recorded as good/poor at booking	Diet quality was presented as good/poor
Pre‐existing physical health conditions	The pre‐existing physical health conditions included in analyses were recorded among women reporting malignancies (e.g., breast cancer), cardiovascular diseases (e.g., hypertension), blood disorders (e.g., anaemia), renal diseases (e.g., congenital renal disease) excluding recurrent Urinary Tract Infections and hepatitis B	Selected pre‐existing physical health conditions were presented as a yes/no measure
Pre‐existing mental health conditions	Pre‐existing severe mental health conditions were recorded among women reporting schizophrenia, bipolar disorder, postnatal psychosis, severe depression, severe eating disorder, severe obsessive‐compulsive disorder and ‘other’	Pre‐existing severe mental health conditions were presented as a yes/no measure.
Previous obstetric complications	Women were recorded as having an obstetric complication in previous pregnancies if reporting at least one obstetric complication at the antenatal stage (e.g., spontaneous miscarriage), delivery stage (e.g., Caesarean section in labour), puerperium stage or based on pregnancy outcomes (e.g., infant died up to 7 days). These data are pre‐populated from entries from previous birth(s)	Previous obstetric complications were presented as a yes/no measure. Only women with a reported gravida ≥ 2 were included.

### 2.3 Statistical Disclosure Control and Analysis

Data were cleaned and prepared for analysis. For example, when necessary to prevent disclosure due to small counts (*n* < 10), certain categories within the variables analysed were combined (e.g., folic acid supplement use responses ‘Missing’ and ‘None’). Numerical restrictions were applied to the BMI indicator: to remove extreme values, it was restricted to include values between 14 and 70 kg/m^2^.

The prevalence of each preconception indicator was calculated. Logistic regression models (including multinomial regression) explored the relationships between each indicator (outcome variables) and area‐level deprivation quintiles (explanatory variable). Both unadjusted models and models adjusted for maternal age group and gravidity (i.e., the number of times a woman had been pregnant, including the current pregnancy) were conducted. Both covariates were introduced simultaneously in the adjusted model. R was used to undertake statistical analyses [20].

### 2.4 Patient and Public Involvement and Engagement

Two members of the Healthy Reproductive Years advisory panel were involved in the application to HBS for data access (e.g., co‐created a ‘plain language’ summary of the proposed study, shaped research questions) and 11 members participated in the prioritisation of the preconception indicators to analyse. Specifically, a prioritisation exercise was carried out on Qualtrics, inviting representatives to rate the importance of 27 preconception indicators on a scale of 1 (less important) to 10 (critically important; Supporting Information S2). These 27 indicators were selected based on their availability within NIMATS. An ‘Unable to score’ option was also available. A final open‐ended question asked respondents to add any further comments. The PPIE activities were reported using the short version of the GRIPP2 checklist (Supporting Information S3) [21].

### 2.5 Terminology

The term ‘woman’ was used throughout the study to also include those who do not identify as women but have been or may become pregnant [15, 22].

## 3 Results

A total of 255 117 pregnancies were eligible for inclusion. Women had a median (interquartile range) age of 30 (8) years. Around a third (31.3%) of pregnancies were primigravida. Results pertaining to the prioritised preconception indicators are presented in Table 2, which also reports the proportion of missing data. Complete case results (i.e., with missing values removed) are shown in Supporting Information S4. Regression models are presented in Tables 3, 4, 5, 6 (Supporting Information S5 includes key figures). The results are presented starting with the indicators with the most robust data quality.

**TABLE 2:** Prevalence of selected preconception indicators in the overall sample.

Preconception indicator	N	%
Deprivation quintiles (*n* = 255 117 pregnancies)		
1, most deprivation	54 621	21.4
2	53 946	21.2
3	52 174	20.5
4	50 513	19.8
5, least deprivation	41 895	16.4
Missing	1968	0.8
Planned pregnancy (*n* = 255 117 pregnancies)		
Yes	180 006	70.6
No	70 686	27.7
Missing	4425	1.7
Smoking (*n* = 255 117 pregnancies)		
Yes	35 194	13.8
No	219 783	86.2
Missing	140	0.1
Folic acid supplement use (*n* = 146 236 pregnancies; from 2014/12/01)		
Preconception 400 μg	48 267	33.0
Preconception 5 mg	6628	4.5
Postconception 400 μg	77 095	52.7
Postconception 5 mg	9224	6.3
None/Missing	5022	3.4
BMI at booking (*n* = 255 117 pregnancies)		
Underweight (< 18.50 kg/m^2^)	4849	1.9
Healthy weight (18.50–24.99 kg/m^2^)	116 686	45.7
Overweight (25.00–29.99 kg/m^2^)	74 397	29.2
With obesity class I (30.00–34.99 kg/m^2^)	33 438	13.1
With obesity class II (35.00–39.99 kg/m^2^)	14 109	5.5
With obesity class III (≥ 40 kg/m^2^)	6809	2.7
Missing	4829	1.9
Alcohol consumption (*n* = 255 117 pregnancies)		
Yes	1332	0.5
No	253 622	99.4
Missing	163	0.1
Diet quality (*n* = 255 117 pregnancies)		
Good	234 429	91.9
Poor	9071	3.6
Missing	11 617	4.6
Pre‐existing physical health conditions (*n* = 255 116 pregnancies)		
Yes	102 631	40.2
No/not reported/missing	152 485	59.8
Pre‐existing severe mental health conditions (*n* = 255 116 pregnancies)		
Yes	35 930	14.1
No/not reported	215 484	84.5
Missing	3702	1.5
Previous obstetric complications, in women with gravidity ≥ 2 (*n* = 175 235 pregnancies)		
Yes	26 224	15.0
No/not reported	149 011	85.0

**TABLE 3:** Associations between deprivation quintiles and planned pregnancy and smoking.

Deprivation quintile	Planned pregnancy		Smoking	
	Unadjusted OR (95% CI)	Adjusted OR (95% CI)	Unadjusted OR (95% CI)	Adjusted OR (95% CI)
1 (most deprived)	**0.33 (0.32–0.34)**	**0.46 (0.45–0.47)**	**5.80 (5.54–6.08)**	**4.18 (3.99–4.38)**
2	**0.57 (0.55–0.59)**	**0.70 (0.67–0.72)**	**3.08 (2.94–3.23)**	**2.53 (2.41–2.65)**
3	**0.69 (0.67–0.71)**	**0.80 (0.77–0.82)**	**2.14 (2.04–2.25)**	**1.84 (1.75–1.93)**
4	**0.79 (0.77–0.82)**	**0.87 (0.84–0.90)**	**1.62 (1.54–1.71)**	**1.47 (1.39–1.55)**
5 (least deprived)	Ref	Ref	Ref	Ref

*Note:* Adjusted: age group + gravidity. Results in bold represent *p* < 0.01.

Abbreviations: CI, confidence interval; OR, odds ratio.

**TABLE 4:** Associations between deprivation quintiles and folic acid supplement use.

Deprivation quintile	Folic acid supplement use (timing)						Folic acid supplement use (dosage)					
	Unadjusted OR (95% CI)			Adjusted OR (95% CI)			Unadjusted OR (95% CI)			Adjusted OR (95% CI)		
	Pre	Post	None	Pre	Post	None	400 μg	5 mg	None	400 μg	5 mg	None
1 (most deprived)	Ref	**2.73 (2.64–2.83)**	**7.07 (6.32–7.90)**	Ref	**2.07 (1.99–2.15)**	**4.38 (3.90–4.90)**	Ref	**1.18 (1.11–1.24)**	**3.86 (3.46–4.30)**	Ref	**1.25 (1.18–1.32)**	**2.78 (2.48–3.11)**
2	Ref	**1.79 (1.72–1.85)**	**3.13 (2.79–3.52)**	Ref	**1.52 (1.47–1.58)**	**2.37 (2.10–2.67)**	Ref	**1.10 (1.04–1.16)**	**2.27 (2.02–2.55)**	Ref	**1.13 (1.07–1.19)**	**1.87 (1.66–2.10)**
3	Ref	**1.51 (1.46–1.56)**	**2.30 (2.04–2.60)**	Ref	**1.33 (1.29–1.38)**	**1.86 (1.64–2.10)**	Ref	**1.08 (1.02–1.14)**	**1.85 (1.64–2.08)**	Ref	**1.10 (1.04–1.16)**	**1.60 (1.42–1.80)**
4	Ref	**1.27 (1.23–1.32)**	**1.70 (1.50–1.93)**	Ref	**1.17 (1.13–1.22)**	**1.49 (1.31–1.69)**	Ref	**1.08 (1.02–1.14)**	**1.51 (1.34–1.71)**	Ref	**1.10 (1.04–1.16)**	**1.38 (1.22–1.57)**
5 (least deprived)	Ref	Ref	Ref	Ref	Ref	Ref	Ref	Ref	Ref	Ref	Ref	Ref

*Note:* Adjusted: age group + gravidity. Results in bold represent *p* < 0.01.

Abbreviations: μg, micrograms; CI, confidence interval; mg, milligrams; OR, odds ratio; Post, postconception; Pre, preconception.

**TABLE 5:** Associations between deprivation quintiles and BMI categories.

Deprivation quintile	BMI categories											
	Unadjusted OR (95% CI)						Adjusted OR (95% CI)					
	Under‐weight	Healthy weight	Over‐weight	With obesity class I	With obesity class II	With obesityclass III	Under‐weight	Healthy weight	Over‐weight	With obesity class I	With obesity class II	With obesity class III
1 (most deprived)	**1.73 (1.57–1.89)**	Ref	**1.20 (1.16–1.21)**	**1.60 (1.53–1.66)**	**1.98 (1.86–2.10)**	**2.38 (2.19–2.60)**	**1.18 (1.07–1.30)**	Ref	**1.27 (1.23–1.31)**	**1.63 (1.56–1.70)**	**2.03 (1.91–2.16)**	**2.52 (2.30–2.75)**
2	**1.22 (1.11–1.35)**	Ref	**1.17 (1.14–1.21)**	**1.45 (1.39–1.51)**	**1.75 (1.65–1.86)**	**1.95 (1.78–2.13)**	0.99 (0.89–1.09)	Ref	**1.20 (1.16–1.24)**	**1.45 (1.39–1.51)**	**1.76 (1.65–1.87)**	**1.98 (1.81–2.17)**
3	1.10 (0.99–1.21)	Ref	**1.16 (1.12–1.19)**	**1.32 (1.27–1.38)**	**1.56 (1.46–1.66)**	**1.73 (1.58–1.89)**	0.94 (0.85–1.03)	Ref	**1.18 (1.14–1.21)**	**1.32 (1.27–1.38)**	**1.56 (1.46–1.66)**	**1.74 (1.59–1.91)**
4	1.10 (0.99–1.21)	Ref	**1.09 (0.99–1.21)**	**1.22 (1.17–1.27)**	**1.35 (1.26–1.43)**	**1.36 (1.24–1.50)**	0.99 (0.90–1.10)	Ref	**1.10 (1.07–1.14)**	**1.22 (1.17–1.27)**	**1.34 (1.26–1.43)**	**1.37 (1.25–1.51)**
5 (least deprived)	Ref	Ref	Ref	Ref	Ref	Ref	Ref	Ref	Ref	Ref	Ref	Ref

*Note:* Adjusted: age group + gravidity. Results in bold represent *p* < 0.01.

Abbreviations: BMI, body mass index; CI, confidence interval; OR, odds ratio.

**TABLE 6:** Associations between deprivation quintiles and alcohol consumption, diet quality, physical health conditions, severe mental health conditions and previous obstetric complications.

Deprivation quintile	Alcohol consumption		Poor diet quality		Physical health conditions		Severe mental health conditions		Previous obstetric complications	
	Unadjusted OR (95% CI)	Adjusted OR (95% CI)	Unadjusted OR (95% CI)	Adjusted OR (95% CI)	Unadjusted OR (95% CI)	Adjusted OR (95% CI)	Unadjusted OR (95% CI)	Adjusted OR (95% CI)	Unadjusted OR (95% CI)	Adjusted^a^ OR (95% CI)
1 (most deprived)	**1.48 (1.25–1.76)**	**1.53 (1.28–1.83)**	**2.41 (2.23–2.60)**	**1.87 (1.73–2.02)**	**1.06 (1.04–1.09)**	**1.10 (1.07–1.13)**	**1.96 (1.89–2.03)**	**1.71 (1.65–1.78)**	**1.14 (1.09–1.20)**	0.96 (0.92–1.01)
2	1.06 (0.88–1.28)	1.09 (0.91–1.32)	**1.75 (1.61–1.89)**	**1.52 (1.40–1.64)**	0.98 (0.96–1.01)	0.99 (0.96–1.02)	**1.35 (1.30–1.41)**	**1.24 (1.20–1.29)**	**1.17 (1.12–1.23)**	**1.06 (1.02–1.11)**
3	0.93 (0.77–1.13)	0.96 (0.79–1.16)	**1.62 (1.50–1.76)**	**1.46 (1.35–1.58)**	**0.97 (0.94–0.99)**	0.97 (0.94–1.00)	**1.19 (1.14–1.24)**	**1.12 (1.07–1.16)**	**1.17 (1.11–1.22)**	**1.08 (1.03–1.13)**
4	1.05 (0.87–1.26)	1.07 (0.89–1.30)	**1.31 (1.20–1.42)**	**1.22 (1.12–1.33)**	**0.95 (0.93–0.98)**	**0.95 (0.93–0.98)**	**1.07 (1.03–1.12)**	1.03 (0.99–1.08)	**1.12 (1.07–1.17)**	**1.06 (1.02–1.11)**
5 (least deprived)	Ref	Ref	Ref	Ref	Ref	Ref	Ref	Ref	Ref	Ref

*Note:* Adjusted: age group + gravidity. Results in bold represent *p* < 0.01.

Abbreviations: CI, confidence interval; OR, odds ratio.

^a^ Adjusted only by age (deprivation quintile + age group), as the indicator is relevant only in women with gravidity ≥ 2.

There was a higher proportion of pregnancies recorded from women in the quintile of greatest deprivation (21.4%) than in the least deprived quintile (16.4%).

Of the pregnancies analysed, 70.6% were recorded as planned. Women living in the most deprived quintile were less likely to report a planned pregnancy than those in the least deprived quintile (Adjusted Odds Ratio [aOR] 0.46, 95% CI 0.45–0.47, *p* < 0.01).

Smoking was recorded in 13.8% of pregnancies, and it was more prevalent among women in the most deprived than in the least deprived quintile (25.7% in q1 and 5.6% in q5). This was mirrored in the regression model, as higher odds of smoking were observed among women in the most deprived quintile relative to the least deprived quintile (aOR 4.18, 95% CI 3.99–4.38, *p* < 0.01).

Preconception supplement use of 400 μg and 5 mg of folic acid was recorded in 33.0% and 4.5% of pregnancies, respectively. Lower proportions were observed for preconception supplement use of 400 μg among women in the most deprived quintile compared to the least deprived quintile (21.4% in q1 and 44.1% in q5). Postconception folic acid supplement use of 400 μg and 5 mg was recorded in 52.7% and 6.3% of pregnancies, respectively, and again lower proportions were observed among women in the most deprived quintile compared to the least deprived quintile (e.g., postconception supplementation of 400 μg: 61.3% in q1 and 44.1% in q5). In the most deprived quintile, there were higher odds of using 5 mg supplements instead of 400 μg (aOR 1.25, 95% CI 1.18–1.32, *p* < 0.01) and of initiating supplement use after conception rather than before (aOR 2.07, 95% CI 1.99–2.15, *p* < 0.01), compared to the least deprived quintile.

Less than half of pregnancies (45.7%) were conceived by women categorised within a healthy BMI category at booking (18.50–24.99 kg/m^2^), and 29.2% and 21.3% of women had overweight (25.00–29.99 kg/m^2^) or obesity (≥ 30.00 kg/m^2^), respectively. There were higher proportions of pregnancies from women with overweight or obesity in the most deprived quintile than the least deprived quintile; for example, 14.7% of pregnancies by women in the most deprived quintile were conceived by women with class I obesity, as opposed to 12.0% in the least deprived quintile. In the regression models, this translated to the odds of living with obesity class I, compared to having a healthy weight, being 63% higher in the most deprived quintile than in the least deprived (aOR 1.63, 95% CI 1.56–1.70, *p* < 0.01).

Self‐reported alcohol consumption was recorded in 0.5% of pregnancies, and prevalence differed based on deprivation quintile (0.7% in q1 and 0.5% in q5). The regression model observed higher odds of alcohol consumption among women in the most deprived quintile relative to the least deprived quintile (aOR 1.53, 95% CI 1.28–1.83, *p* < 0.01).

Women's self‐reported diet quality was recorded as ‘good’ in 91.9% of pregnancies. More women in the most deprived quintile reported consuming a poor quality diet than women in the least deprived quintile (5.1% in q1 and 2.2% in q5; aOR 1.87, 95% CI 1.73–2.02, *p* < 0.01).

Physical health and severe mental health conditions were recorded in 40.2% and 14.1% of pregnancies, respectively. Compared to the least deprived quintile, women in the most deprived quintile had higher odds of reporting at least one physical health or severe mental health condition (aOR 1.10, 95% CI 1.07–1.13, *p* < 0.01 and aOR 1.71, 95% CI 1.65–1.78, *p* < 0.01, respectively).

Among women with gravidity ≥ 2, previous obstetric complications (e.g., spontaneous miscarriage, Caesarean section in labour) were recorded in 15.0% of pregnancies. The lowest proportion was recorded for pregnancies conceived by women from the least deprived quintile (15.2% in q1 and 13.5% in q5), although when comparing odds in these two groups, the results did not reach statistical significance.

### 3.1 Patient and Public Involvement and Engagement

The results of the prioritisation exercise (reported in full in Supporting Information S2) informed the methods of the study. Of note, based on the average score each indicator received (1 referred to less important and 10 to critically important), the indicators that were considered the most important were smoking and alcohol consumption (average score: 9.36), followed by general health indicators such as blood sugar and blood pressure (average score: 8.73), diet quality and previous surgical history (average score: 8.64) and pre‐existing health conditions and previous obstetric history (average score: 8.55). Additional relevant indicators were mentioned (e.g., weight stigma, financial security, disabilities) which were not prioritised.

## 4 Discussion

### 4.1 Main Findings

This study described the prevalence of key preconception health indicators recorded in NIMATS (2011–2021) and explored differences based on maternal area‐level deprivation in over 255 000 pregnancies. The findings suggested that many risk factors were common; for example, over 50% of pregnancies were conceived by women with underweight, overweight, or obesity, and more than 60% by women with sub‐optimal preconception folic acid supplement use. Women living in the least deprived area generally reported better preconception health indicators than women in the other deprivation areas.

### 4.2 Interpretation

Similar to our findings, preconception health disparities have been reported elsewhere [9, 23, 24, 25, 26]. For example, data from England from 2018 to 2019 suggested that women in the most deprived quintile were more likely to report not taking preconception folic acid supplements compared to women in the least deprived quintile (83.0% vs. 60.6%) [23]. Additionally, stillbirth and neonatal mortality rates are higher for offspring born to women from the most deprived areas than the least deprived [27]. These significant disparities in preconception health highlight a need for resource allocation in deprived areas and co‐development of strategies with women in these areas to address preconception, maternal and infant inequalities [3, 28]. Notably, this finding presents a crucial opportunity for policymakers to focus on these at‐risk communities through preventative measures and interventions aimed at reducing longstanding health disparities in NI. However, the inter‐relationship between deprivation and health outcomes is highly complex and also associated with other factors, such as ethnicity and engagement with the healthcare system [3, 9, 29], not investigated in this study.

The recorded prevalence of self‐reported smoking (13.8%) was lower than that recorded for England (19.5%) [9] and other countries such as Italy (26.4%) [30] and the US (19.4%) [31]. Given the known harms of smoking on conception, greater efforts to improve this indicator, such as enhanced support services, are warranted. Although not currently reported in NIMATS, the use of e‐cigarettes should be considered as these are increasingly used among adults in NI [32] and may impact (reproductive) health [33, 34].

Preconception folic acid supplement use was recorded in 37.5% of pregnancies. However, it was not possible to ascertain whether women who reported supplement use adhered to the recommendation to start daily use at least 12 weeks before conception [35]. For women who reported postconception use, the exact timing of initiation (e.g., shortly after conception, or just before their antenatal appointment) could not be determined. Given the importance of adequate folic acid supplement use before conception, inclusive and effective efforts should be made to improve use in NI.

The analysis of BMI at booking showed that 29.2% of pregnancies were conceived by women living with overweight and 21.3% by women living with obesity. Similar proportions have been recorded in other countries across the UK [36]. Because women with obesity have a higher risk of many pregnancy‐related complications compared to women with a healthy BMI [37], this indicator warrants particular attention. Multi‐level systems approaches are needed to prevent obesity, whilst also supporting those already with obesity, to minimise health risks and reduce the impact of intergenerational transmission.

Certain findings from this study require careful interpretation, due to the data used. Notably, most pregnancies were self‐reported as planned (70.6%), echoing a previous study in NI (72.4%) [14]. However, data collection for this indicator could be subject to social desirability and lacks validation. The London Measure of Unplanned Pregnancy, a validated tool [38] already successfully implemented across certain parts of England [39] and Australia [40], could be used going forward to assess pregnancy planning and behavioural preparation more comprehensively than the current NIMATS measure. Moreover, results pertaining to alcohol drinking (0.5%) reflects consumption during the early stages of pregnancy, rather than before conception, and may be influenced by social desirability bias [41]. Additionally, results pertaining to ‘good’ diet quality (91.9%) contradict national dietary surveys which detail many areas where dietary recommendations are not being met (e.g., fibre, fruit and vegetable consumption, free sugars intake) [42]. This disparity likely reflects a limitation of the binary question used in NIMATS to assess dietary intake (‘good/poor’). Overall, results suggest that the methods of recording preconception alcohol consumption and diet quality at antenatal appointments should be reviewed.

Pre‐existing physical health condition(s) and severe mental health condition(s) were recorded in 40.2% and 14.1% of pregnancies, respectively. In comparison, English data from 2018 to 2019 indicated that 24.3% of women had at least one known pre‐existing mental or physical health condition [9], and UK data from 2018 found a multimorbidity prevalence of 44.2% [43]. These different findings observed between countries may be influenced by the different sources and methods of routine data collection. The importance of interconception care is also highlighted in this study. However, due to the quality of these data, findings should be interpreted with caution.

### 4.3 Patient and Public Involvement and Engagement

The PPIE representatives considered smoking, alcohol intake, general health factors, and (previous) medical history (e.g., surgical history) the most important preconception indicators, with maternal sociodemographic factors not generally considered a high priority. This may reflect respondents' belief that these factors have a less direct impact on maternal and child health outcomes than other factors.

### 4.4 Strengths and Limitations

This study contributes to the current epidemiological understanding of preconception health and fills an important gap in the characterisation of women's preconception health in NI. Partnering with PPIE representatives ensured relevance of the research and informed the prioritisation of indicators.

Some variables were self‐reported to HCPs during booking appointments (e.g., diet quality, planned pregnancy) and may be influenced by over‐ or under‐reporting and social desirability bias. Adding simple validated tools could enhance data quality and reduce variability in how HCPs frame questions when collecting data, which can also influence how women respond. Alongside these improvements, it is important to ensure that HCPs have sufficient time and skills to record comprehensive data.

Overall, the scope of analysis was limited by the availability of data within NIMATS. This study analysed preconception indicators based on area‐level deprivation quintiles, not taking into account other social determinants of health due to limited data availability. Focusing on intersectionality and key patient characteristics such as ethnicity, where data permit, may provide a greater understanding of women's health in NI, though the limited ethnic diversity of the country should be considered. Indeed, approximately 10% of births in NI are from mothers born outside the UK or Ireland, and less than 4% of the general population belong to an ethnic minority group [44, 45, 46]. Additionally, because deprivation was area‐level, rather than specific to each individual, in some circumstances an under‐ or over‐estimation of deprivation may have occurred (e.g., ‘affluent’ households being grouped within areas of deprivation). Finally, while this study exclusively focused on women, the preconception health of men also warrants investigation [47].

## 5 Conclusion

This study presents a national overview of preconception health among women in NI as recorded in routinely‐collected maternity data (2011–2021). It provides a reference point for emerging efforts to improve and reduce inequalities in preconception health and care, pregnancy outcomes and the health of future offspring. It also highlights areas warranting particular focus, such as sub‐optimal preconception folic acid supplement use and high levels of obesity, especially among women living in the most deprived areas. Given the limitations identified in the data used, an opportunity to improve the data quality of routinely‐collected preconception health indicators was identified.

## Author Contributions

E.H.C., L.M., L.K., M.C.M., D.A.J.M.S. and K.‐A.E. contributed to the conceptualisation and planning of the research and the access to the data used. E.H.C., L.M. and L.K., who had direct access to the data, analysed the data presented in the manuscript, with input on methodology from other co‐authors. E.H.C. drafted the manuscript, which was finalised with feedback provided by L.M., L.K., M.C.M., D.A.J.M.S. and K.‐A.E. All authors have seen and approved the final version of the manuscript for publication.

## Ethics Statement

Ethical approval and participant consent were not required as the study used previously held secondary data, de‐identified at the point of access. Researchers completed the Safe Researcher training provided by the Office for National Statistics to be eligible to access the data and undertake the study.

## Conflicts of Interest

The authors declare no conflicts of interest.

## Supporting information


Data S1.

## Data Availability

The data that support the findings of this study are available from the Honest Broker Service (HBS) within the Business Services Organisation Northern Ireland (BSO) following a successful application.

## References

[1] C. L. Robbins , D. D'Angelo , L. Zapata , et al., “Preconception Health Indicators for Public Health Surveillance,” Journal of Women's Health 27, no. 4 (2018): 430–443. 10.1089/jwh.2017.6531 PMC5903944 29323604

[2] World Health Organization , “Women of Reproductive Age (15–49 Years) Population (Thousands),” (2024), https://platform.who.int/data/maternal‐newborn‐child‐adolescent‐ageing/indicator‐explorer‐new/MCA/women‐of‐reproductive‐age‐(15‐49‐years)‐population‐(thousands).

[3] Public Health England , “Making the Case for Preconception Care,” (2018), https://assets.publishing.service.gov.uk/government/uploads/system/uploads/attachment_data/file/729018/Making_the_case_for_preconception_care.pdf.

[4] C. Mazzali and P. Duca , “Use of Administrative Data in Healthcare Research,” Internal and Emergency Medicine 10 (2015): 517–524. 25711312 10.1007/s11739-015-1213-9

[5] L. L. Nguyen and N. R. Barshes , “Analysis of Large Databases in Vascular Surgery,” Journal of Vascular Surgery 52, no. 3 (2010): 768–774. 20598475 10.1016/j.jvs.2010.03.027

[6] D. A. Grimes , “Epidemiologic Research Using Administrative Databases: Garbage in, Garbage out,” Obstetrics and Gynecology 116, no. 5 (2010): 1018–1019. 20966682 10.1097/AOG.0b013e3181f98300

[7] L. L. Lipscombe and J. E. Hux , “Trends in Diabetes Prevalence, Incidence, and Mortality in Ontario, Canada 1995–2005: A Population‐Based Study,” Lancet 369, no. 9563 (2007): 750–756. 17336651 10.1016/S0140-6736(07)60361-4

[8] J. Stephenson , C. Vogel , J. Hall , et al., “Preconception Health in England: A Proposal for Annual Reporting With Core Metrics,” Lancet 393, no. 10187 (2019): 2262–2271. 31162084 10.1016/S0140-6736(19)30954-7

[9] D. A. J. M. Schoenaker , J. Stephenson , H. Smith , et al., “Women's Preconception Health in England: A Report Card Based on Cross‐Sectional Analysis of National Maternity Services Data From 2018/2019,” BJOG: An International Journal of Obstetrics and Gynaecology 130, no. 10 (2023): 1187–1195. 36810878 10.1111/1471-0528.17436 PMC10952348

[10] B. H. A. Groot , M. Buree , R. V. Zuijlen , et al., “What Patients Prioritize for Research to Improve Their Lives and How Their Priorities Get Dismissed Again,” International Journal of Environmental Research and Public Health 19, no. 4 (2022): 1927. 35206113 10.3390/ijerph19041927 PMC8871903

[11] P. Raval , F. Moreno , and I. Needleman , “Patient Involvement to Explore Research Prioritisation and Self‐Care Management in People With Periodontitis and Diabetes,” British Dental Journal 5 (2021): 9. 10.1038/s41415-021-3175-9 34239054

[12] D. L. Broussard , W. B. Sappenfield , C. Fussman , C. D. Kroelinger , and V. Grigorescu , “Core State Preconception Health Indicators: A Voluntary, Multi‐State Selection Process,” Maternal and Child Health Journal 15, no. 2 (2011): 158–168. 20225127 10.1007/s10995-010-0575-x

[13] D. A. J. M. Schoenaker , J. Stephenson , A. Connolly , et al., “Characterising and Monitoring Preconception Health in England: A Review of National Population‐Level Indicators and Core Data Sources,” Journal of Developmental Origins of Health and Disease 13, no. 2 (2021): 137–150. 34085623 10.1017/S2040174421000258 PMC7612507

[14] L. Kent , C. Cardwell , I. Young , and K.‐A. Eastwood , “Trends in Maternal Body Mass Index in Northern Ireland: A Cross‐Sectional and Longitudinal Study,” Family Medicine and Community Health 9, no. 4 (2021): e001310. 34949675 10.1136/fmch-2021-001310 PMC8710425

[15] National Institute for Health and Care Excellence , “Antenatal Care,” (2021), http://www.nice.org.uk/guidance/ng201.

[16] National Health Service , “Your Antenatal Appointments,” (2023), https://www.nhs.uk/pregnancy/your‐pregnancy‐care/your‐antenatal‐appointments/#:~:text=8%20to%2012%20weeks%3A%20booking,you're%2010%20weeks%20pregnant.

[17] J. Ijpelaar , T. Power , and B. Green , “Northern Ireland Multiple Deprivation Measures 2017,” Journal of the Statistical and Social Inquiry Society of Ireland 48 (2020): 163–174.

[18] Northern Ireland Statistics and Research Agency , “Super Output Areas (Census 2011),” https://nisra.gov.uk/support/output‐geography‐census‐2011/super‐output‐areas.

[19] NICE , “Obesity: Identification, Assessment and Management,” (2014), https://www.nice.org.uk/guidance/cg189. 36719951

[20] R Core Team , “R: A Language and Environment for Statistical Computing,” (2022), https://www.R‐project.org.

[21] S. Staniszewska , J. Brett , I. Simera , et al., “GRIPP2 Reporting Checklists: Tools to Improve Reporting of Patient and Public Involvement in Research,” BMJ 358 (2017): j3453. 28768629 10.1136/bmj.j3453 PMC5539518

[22] National Institute for Health and Care Excellence , “Postnatal Care,” (2021), https://www.nice.org.uk/guidance/ng194.

[23] Office for Health Improvement and Disparities , “Report Card: Indicators of Women's Preconception Health,” (2022), https://www.gov.uk/government/publications/report‐card‐indicators‐of‐womens‐preconception‐health.

[24] R. Terry , A. Gatewood , C. Elenwo , et al., “Disparities in Preconception Health Indicators in U.S. Women: A Cross‐Sectional Analysis of the Behavioral Risk Factor Surveillance System 2019,” Journal of Perinatal Medicine 52, no. 2 (2024): 192–201. 38146265 10.1515/jpm-2023-0249 PMC11190353

[25] W. Momino , L. Minussi , D. Woffchuck , et al., “Reproductive Risk Factors Related to Socioeconomic Status in Pregnant Women in Southern Brazil,” Community Genetics 6, no. 2 (2003): 77–83. 14560067 10.1159/000073002

[26] H. Rowe , S. Holton , M. Kirkman , et al., “Prevalence and Distribution of Unintended Pregnancy: The Understanding Fertility Management in Australia National Survey,” Australian and New Zealand Journal of Public Health 40, no. 2 (2016): 104–109. 26456762 10.1111/1753-6405.12461

[27] I. D. Gallimore , R. J. Matthews , G. L. Page , et al., “MBRRACE‐UK Perinatal Mortality Surveillance: UK Perinatal Deaths of Babies Born in 2022,” https://timms.le.ac.uk/mbrrace‐uk‐perinatal‐mortality/surveillance/files/MBRRACE‐UK‐perinatal‐mortality%20surveillance‐report‐2022.pdf.

[28] D. Schoenaker , J. Hall , C. Stewart , et al., “Tackling Inequalities in Preconception Health and Care: Barriers, Facilitators and Recommendations for Action From the 2023 UK Preconception EMCR Network Conference,” Journal of Developmental Origins of Health and Disease 15 (2024): e24. 39444318 10.1017/S204017442400031X

[29] O. Ojukwu , D. Patel , J. Stephenson , B. Howden , and J. Shawe , “General Practitioners' Knowledge, Attitudes and Views of Providing Preconception Care: A Qualitative Investigation,” Upsala Journal of Medical Sciences 121, no. 4 (2016): 256–263. 27646963 10.1080/03009734.2016.1215853 PMC5098490

[30] P. Mastroiacovo , R. M. Nilsen , E. Leoncini , et al., “Prevalence of Maternal Preconception Risk Factors: An Italian Multicenter Survey,” Italian Journal of Pediatrics 40, no. 1 (2014): 91. 25416843 10.1186/s13052-014-0091-5 PMC4264313

[31] J. E. Anderson , S. Ebrahim , L. Floyd , and H. Atrash , “Prevalence of Risk Factors for Adverse Pregnancy Outcomes During Pregnancy and the Preconception Period—United States, 2002–2004,” Maternal and Child Health Journal 10, no. S1 (2006): 101–106. 10.1007/s10995-006-0093-z PMC1592157 16710762

[32] D. Corrigan , M. Scarlett , and B. Stewart , “Health Survey (NI)—First Results 2022/23,” (2023), https://www.health‐ni.gov.uk/sites/default/files/publications/health/hsni‐first‐results‐22‐23.pdf.

[33] K. Szumilas , P. Szumilas , A. Grzywacz , and A. Wilk , “The Effects of e‐Cigarette Vapor Components on the Morphology and Function of the Male and Female Reproductive Systems: A Systematic Review,” International Journal of Environmental Research and Public Health 17, no. 17 (2020): 6152. 32847119 10.3390/ijerph17176152 PMC7504689

[34] R. Sahu , K. Shah , R. Malviya , et al., “E‐Cigarettes and Associated Health Risks: An Update on Cancer Potential,” Advances in Respiratory Medicine 91, no. 6 (2023): 516–531. 37987300 10.3390/arm91060038 PMC10660480

[35] National Health Service , “How and When to Take Folic Acid,” (2022), https://www.nhs.uk/medicines/folic‐acid/how‐and‐when‐to‐take‐folic‐acid/.

[36] NMPA Project Team , “National Maternity and Perinatal Audit: Clinical Report 2017,” (2018), https://maternityaudit.org.uk/downloads/NMPA%20Clinical%20Report%202018.pdf.

[37] F. Denison , N. Aedla , O. Keag , et al., “Care of Women With Obesity in Pregnancy,” BJOG: An International Journal of Obstetrics & Gynaecology 126, no. 3 (2019): e62–e106. 30465332 10.1111/1471-0528.15386

[38] J. A. Hall , G. Barrett , A. Copas , and J. Stephenson , “London Measure of Unplanned Pregnancy: Guidance for Its Use as an Outcome Measure,” Patient Related Outcome Measures 8 (2017): 43–56. 28435343 10.2147/PROM.S122420 PMC5388237

[39] J. A. Hall , C. Stewart , B. Stoneman , et al., “Implementation of the London Measure of Unplanned Pregnancy in Routine Antenatal Care: A Mixed‐Methods Evaluation in Three London NHS Trusts,” European Journal of Midwifery 8 (2024): 1–13. 10.18332/ejm/188118 PMC11145722 38832251

[40] K. I. Black , E. Dorney , J. A. Hall , M. Pelosi , S. A. Khan , and K. Cheney , “Using a Validated Instrument to Assess Pregnancy Planning and Preconception Care at Antenatal Booking Visits: A Retrospective Cohort Study,” Medical Journal of Australia 219, no. 8 (2023): 366–370. 37743071 10.5694/mja2.52109

[41] L. M. O'Keeffe , P. M. Kearney , F. P. McCarthy , et al., “Prevalence and Predictors of Alcohol Use During Pregnancy: Findings From International Multicentre Cohort Studies,” BMJ Open 5, no. 7 (2015): e006323. 10.1136/bmjopen-2014-006323 PMC4499685 26152324

[42] Food Standards Agency in Northern Ireland, Public Health England , “National Diet and Nutrition Survey (NDNS) Report for Northern Ireland,” (2019), https://www.food.gov.uk/research/national‐diet‐and‐nutrition‐survey/national‐diet‐and‐nutrition‐survey‐ndns‐report‐for‐northern‐ireland.

[43] S. I. Lee , A. Azcoaga‐Lorenzo , U. Agrawal , et al., “Epidemiology of Pre‐Existing Multimorbidity in Pregnant Women in the UK in 2018: A Population‐Based Cross‐Sectional Study,” BMC Pregnancy and Childbirth 22, no. 1 (2022): 120. 35148719 10.1186/s12884-022-04442-3 PMC8840793

[44] Northern Ireland Statistics and Research Agency , “Registrar General Annual Report 2022 Births,” (2023), https://www.nisra.gov.uk/publications/registrar‐general‐annual‐report‐2022‐births.

[45] Northern Ireland Statistics and Research Agency , “Main Statistics for Northern Ireland Statistical Bulletin: Ethnic Group,” (2022), https://www.nisra.gov.uk/system/files/statistics/census‐2021‐main‐statistics‐for‐northern‐ireland‐phase‐1‐statistical‐bulletin‐ethnic‐group.pdf.

[46] Northern Ireland Statistics and Research Agency , “Census 2021 Main Statistics Ethnicity Tables,” (2022), https://www.nisra.gov.uk/publications/census‐2021‐main‐statistics‐ethnicity‐tables.

[47] T. Carter , D. Schoenaker , J. Adams , and A. Steel , “Paternal Preconception Modifiable Risk Factors for Adverse Pregnancy and Offspring Outcomes: A Review of Contemporary Evidence From Observational Studies,” BMC Public Health 23, no. 1 (2023): 509. 36927694 10.1186/s12889-023-15335-1 PMC10022288

