# Supplementary material for: Preconception Health Indicators and Deprivation: A Cross‐Sectional Study Using National Maternity Healthcare Data
Source: BJOG. 2025 Jun 19;132(13):2138–48. doi: 10.1111/1471-0528.18256 (PMC12592756; doi:10.1111/1471-0528.18256)
Supplement: Supplementary file 1 — Data S1. [file BJO-132-2138-s001.docx]

# Supplementary Information S1

Table 1. Details of 10 priority indicators, including additional comments from a midwife consultant and data quality considerations

| ***Indicator*** | ***How is it recorded in NIMATS?*** | ***Additional comments from midwife consultant*** | ***How is it presented in this study?*** | ***Data quality***  ***interpretation*** |
| --- | --- | --- | --- | --- |
| Deprivation quintile* | Area-level deprivation was estimated using NIMDM 2017, which aggregates the ranking of seven specific domains into a single ranking. These weighted domains are: 25% income, 25% employment, 15% health and disability, 15% education, skills and training, 10% access to services, 5% living environment, and 5% crime and disorder^1^. Maternal data in NIMATS were linked to the NIMDM by the super output area code^2^. | / | Area-level deprivation was categorised into quintiles, where the first quintile represented the most deprived areas and the fifth the least deprived ones. | + (indicates higher confidence in data quality, e.g. objective measure etc.) |
| Planned pregnancy | Planned pregnancies were recorded as a binary Yes/No measure at booking. | Potentially framed as ‘Was this a planned pregnancy?’ | Planned pregnancies were presented as a Yes/No measure. | + |
| Smoking | Smoking status was calculated based on the reported number of cigarettes smoked per day at booking. | Measures of carbon monoxide levels at booking can provide additional insights. | Smoking status was presented as a Yes/No measure. | + |
| Folic acid supplement use | Folic acid supplement use was categorised based on both the timing (i.e., preconception and postconception) and dose reported (i.e., 400μg or 5mg). The data included for this measure were only pertaining to pregnancies with a booking appointment date after 01/12/2014, because of changes in the recording in NIMATS. | / | Folic acid supplement use was presented as Preconception 400μg, Preconception 5mg, Postconception 400μg, Postconception 5mg, and None. | + |
| BMI | BMI was derived by clinically measured weight and height recorded at booking. | / | Women were divided into: underweight (<18.49 kg/m^2^), healthy weight (18.50–24.99 kg/m^2^), overweight (25.00–29.99 kg/m^2^), with obesity class I (30.00–34.99 kg/m^2^), with obesity class II (35.00–39.99 kg/m^2^), and with obesity class III (≥40.00 kg/m^2^)^3^. Recordings of BMI were included if in the range of 14-70 kg/m^2^. | + |
| Alcohol consumption | Alcohol consumption was calculated based on the reported units of alcohol consumed per week at booking. | Potentially framed as ‘Have you consumed alcohol in your pregnancy?’ | Alcohol consumption was presented as a Yes/No measure. | ~ (data quality requires caution in the interpretation, e.g. due to subjectivity, framing of questions, or issues with data entry) |
| Diet quality | Diet quality was recorded as Good/Poor at booking. | Generally framed as ‘Do you eat a varied diet?’ | Diet quality was presented as Good/Poor. | ~ |
| Pre-existing physical health conditions | The pre-existing physical health conditions included in analyses were recorded among women reporting malignancies (e.g., breast cancer), cardiovascular diseases (e.g., hypertension), blood disorders (e.g., anaemia), renal diseases (e.g., congenital renal disease) excluding recurrent UTIs, and hepatitis B. | Healthcare professionals usually systematically inquire about physical health conditions from a broad list that drops down into certain specific conditions.  Some women may be referred by a GP, meaning they will have a referral letter in their notes. This provides a medical summary that healthcare professionals can check with the woman. | Selected pre-existing physical health conditions were presented as a Yes/No measure. | ~ |
| Pre-existing mental health conditions | Pre-existing severe mental health conditions were recorded among women reporting schizophrenia, bipolar disorder, postnatal psychosis, severe depression, severe eating disorder, severe obsessive-compulsive disorder, and ‘other’. | Healthcare professionals usually systematically inquire about mental health conditions from a broad list that drops down into certain specific conditions.  Some women may be referred by a GP, meaning they will have a referral letter in their notes. This provides a medical summary that healthcare professionals can check with the woman.  If women disclose they are under the care of a mental health team, healthcare professionals may liaise with relevant professionals (e.g., community psychiatric nurses). | Pre-existing severe mental health conditions were presented as a Yes/No measure. | ~ |
| Previous obstetric complications | Women were reported as having an obstetric complication in previous pregnancies if reporting at least one obstetric complication at the antenatal stage (e.g., spontaneous miscarriage), delivery stage (e.g., Caesarean section in labour), puerperium stage or based on pregnancy outcomes (e.g., infant died up to 7 days). These data are pre-populated from entries from previous birth(s). | The measure may be prepopulated from previous entries from the woman’s last birth if available. | Previous obstetric complications were presented as a Yes/No measure. Only women with a reported gravida ≥2 were included. | ~ |
| *Not recorded in NIMATS; postcodes were used to discern deprivation quintiles. Abbreviations: BMI: Body Mass Index; GP: General Practitioner; kg: kilogram; m: metre; mg: milligram; NIMATS: Northern Ireland Maternity System; NIMDM: Northern Ireland Multiple Deprivation Measure; UTI: Urinary Tract Infection; μg: microgram. | | | | |

# Supplementary Information S2

Table 1. Average score for each of the preconception indicators included in the prioritisation exercise directed at the Healthy Reproductive Years Patient and Public Involvement and Engagement (PPIE) panel (n=11).

| ***Indicator heading*** | ***Indicator*** | ***Average score (1-10)**** |
| --- | --- | --- |
| About the mum | Mum’s age at booking | 6.64 |
|  | Mum’s financial hardship/deprivation | 6.36 |
|  | Mum requires a translator | 6.10 |
|  | Mum sees a social worker | 5.45 |
|  | Mum’s partner is present at the antenatal booking appointment | 4.82 |
|  | Mum’s employment (Employed, Unemployed) | 4.64 |
|  | Mum’s marital status (Divorced/Separated, Married/Civil Partner/Common Law, Single, Other) | 4.09 |
|  | Mum’s ethnicity and country of birth (UK, Other) | 3.40 |
| About the mum’s behaviours | Mum’s smoking behaviour (if the mum currently smokes, if the mum has been referred to smoking cessation services, if the mum has received tailored advice on smoking) | 9.36 |
|  | Mum’s consumption of alcohol | 9.36 |
|  | Mum’s diet quality (good or poor) | 8.64 |
|  | Mum’s use of medicines | 8.45 |
|  | Mum’s folic acid supplementation (to prevent neural tube defects in babies) | 8.36 |
|  | Mum looked into or received treatment to assist fertility (e.g., In vitro fertilisation, Egg donation, Sperm donation etc.) | 7.64 |
|  | Information on mum’s sleep pattern | 7.64 |
|  | Mum’s weight at booking, in relation to their height (Body Mass Index) | 7.45 |
|  | Mum planned the pregnancy | 7.36 |
|  | Routine vaccines received (Rubella, whooping cough, flu) | 6.55 |
|  | Mum’s most recent contraception (e.g., pill, condom, patch, etc.) | 6.18 |
|  | Other vaccines received, including sexual health vaccines (e.g., Hepatitis B, Syphilis) | 6.18 |
| About the mum’s health | Mum’s general health (e.g., blood sugar, blood pressure) | 8.73 |
|  | Information about mum’s previous surgical history (e.g., surgery relating to gynaecology) | 8.64 |
|  | Information about mum’s pre-existing medical health conditions (e.g., epilepsy, diabetes) | 8.55 |
|  | Information about mum’s previous obstetric history (i.e., relative to previous pregnancies) and general sexual health | 8.55 |
|  | Information about mum’s mental health (e.g., does the mum have anxiety?) and mood (e.g., has the mum often been bothered by having little interest or pleasure in doing things?) | 8.10 |
|  | Mum’s history of domestic violence | 7.82 |
|  | Information about mum’s family health history (e.g., tuberculosis recorded as part of the mum’s family history) | 7.55 |
| *Respondents rated the importance of each preconception indicator on a scale from 1 (less important) to 10 (critically important); an ‘Unable to score’ option was provided. | | |

# Supplementary Information S3

Table 1. Guidance for Reporting Involvement of Patients and the Public (GRIPP) short-form checklist.

| ***Section and topic*** | ***Item*** | ***Reported on page No*** |
| --- | --- | --- |
| 1: Aim | Report the aim of PPIE in the study | 8 |
| 2: Methods | Provide a clear description of the methods used for PPIE in the study | 8 |
| 3. Study results | Outcomes—Report the results of PPIE in the study, including both positive and negative outcomes | 10-11 |
| 4. Discussion and conclusion | Outcomes—Comment on the extent to which PPIE influenced the study overall. Describe positive and negative effects | 13-14 |
| 5. Reflections/critical perspective | Comment critically on the study, reflecting on the things that went well and those that did not, so others can learn from this experience | 13-14 |
| Abbreviation: PPIE Patient and Public Involvement and Engagement. | | |

# Supplementary Information S4

Table 1. Prevalence of selected preconception indicators in the overall sample (complete cases).

| ***Preconception indicator**** | ***% (complete cases)*** |
| --- | --- |
| Deprivation quintiles (n= 253,149 pregnancies) | |
| - 1, most deprivation | 21.58 |
| - 2 | 21.31 |
| - 3 | 20.61 |
| - 4 | 19.95 |
| - 5, least deprivation | 16.55 |
| Planned pregnancy (n= 250,692pregnancies) | |
| - Yes | 71.8 |
| - No | 28.2 |
| Smoking (n= 254,997 pregnancies) | |
| - Yes | 13.8 |
| - No | 86.2 |
| BMI at booking (n= 250,288 pregnancies) | |
| - Underweight (<18.5 kg/m2) | 1.94 |
| - Healthy weight (18.5–24.99 kg/m2) | 46.62 |
| - Overweight (25.00–29.99 kg/m2) | 29.72 |
| - With obesity class I (30.00–34.99 kg/m2) | 13.36 |
| - With obesity class II (35.00–39.99 kg/m2) | 5.64 |
| - With obesity class III (≥40 kg/m^2^) | 2.72 |
| Alcohol consumption (n= 254,954 pregnancies) | |
| - Yes | 0.52 |
| - No | 99.48 |
| Diet quality (n= 243,500 pregnancies) | |
| - Good | 96.27 |
| - Poor | 3.73 |
| Severe pre-existing mental health conditions (n= 251,414 pregnancies) | |
| - Yes | 14.29 |
| - No/Not reported | 85.71 |
| *Folic acid supplement use is not included to comply with disclosure controls (missing values n<10). However, no differences in the proportions were detected. Similarly, physical health conditions and previous obstetric complications in women with gravida ≥ 2 are not presented. | |

| Supplementary Information S5 |  | |  |
| --- | --- | --- | --- |
| Figure 1a. Prevalence of reported planned pregnancy based on deprivation quintile. | Figure 1b. Prevalence of reported smoking based on deprivation quintile. | |  |
| Figure 1c. Prevalence of reported folic acid supplement use levels based on deprivation quintile. | | Figure 1d. Prevalence of BMI categories based on deprivation quintile. | |
| Figure 1e. Prevalence of reported alcohol consumption based on deprivation quintile. | | Figure 1f. Prevalence of reported diet quality based on deprivation quintile. | |
| Figure 1g. Prevalence of reported physical health conditions based on deprivation quintile. | | Figure 1h. Prevalence of reported severe mental health conditions based on deprivation quintile. | |
| Figure 1i. Prevalence of previous obstetric complications (in women with gravida ≥2) based on deprivation quintile. | | | |

# Supplementary Information S5


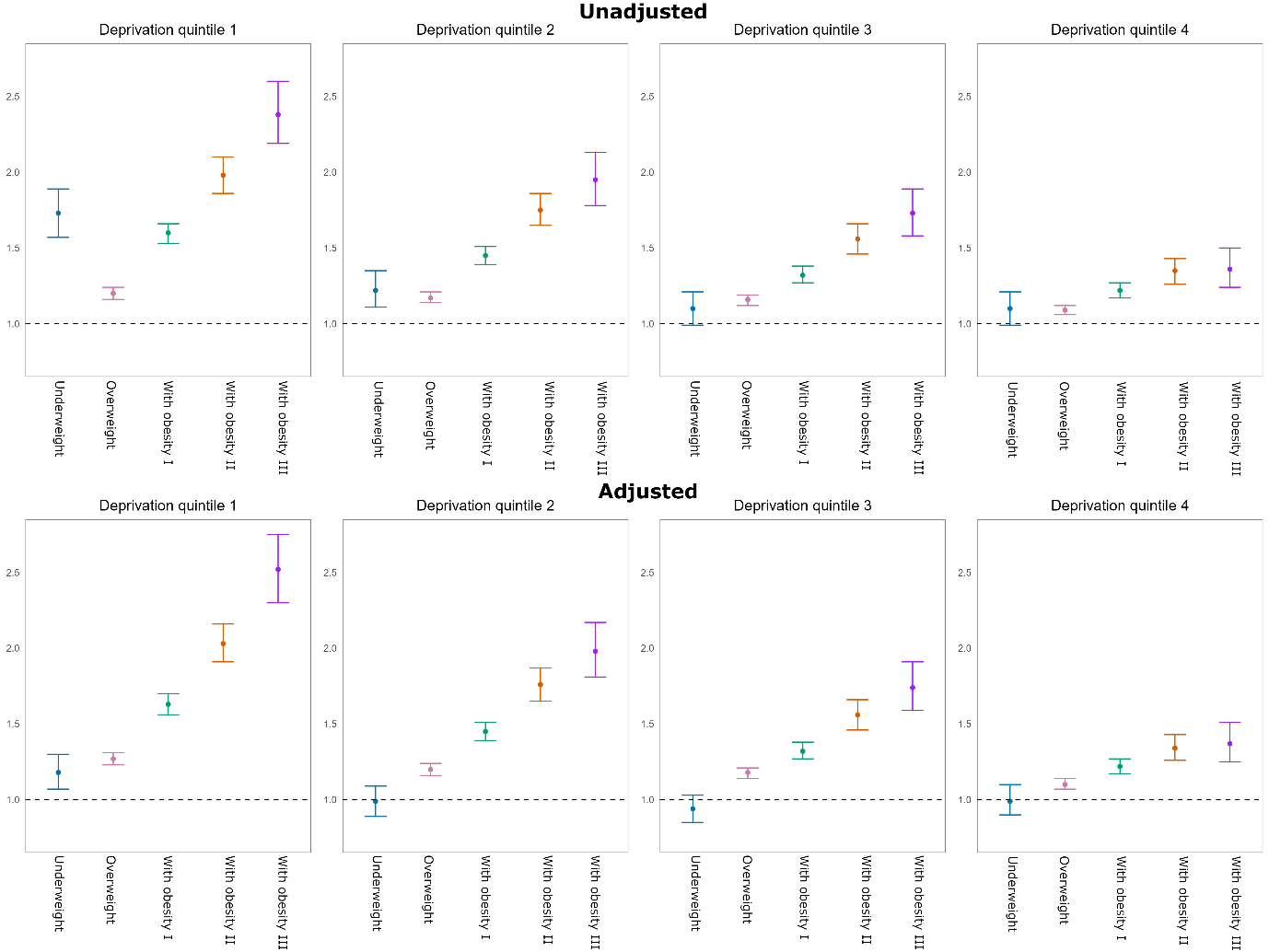


Figure 1. Deprivation vs BMI categories: OR (95%CI)


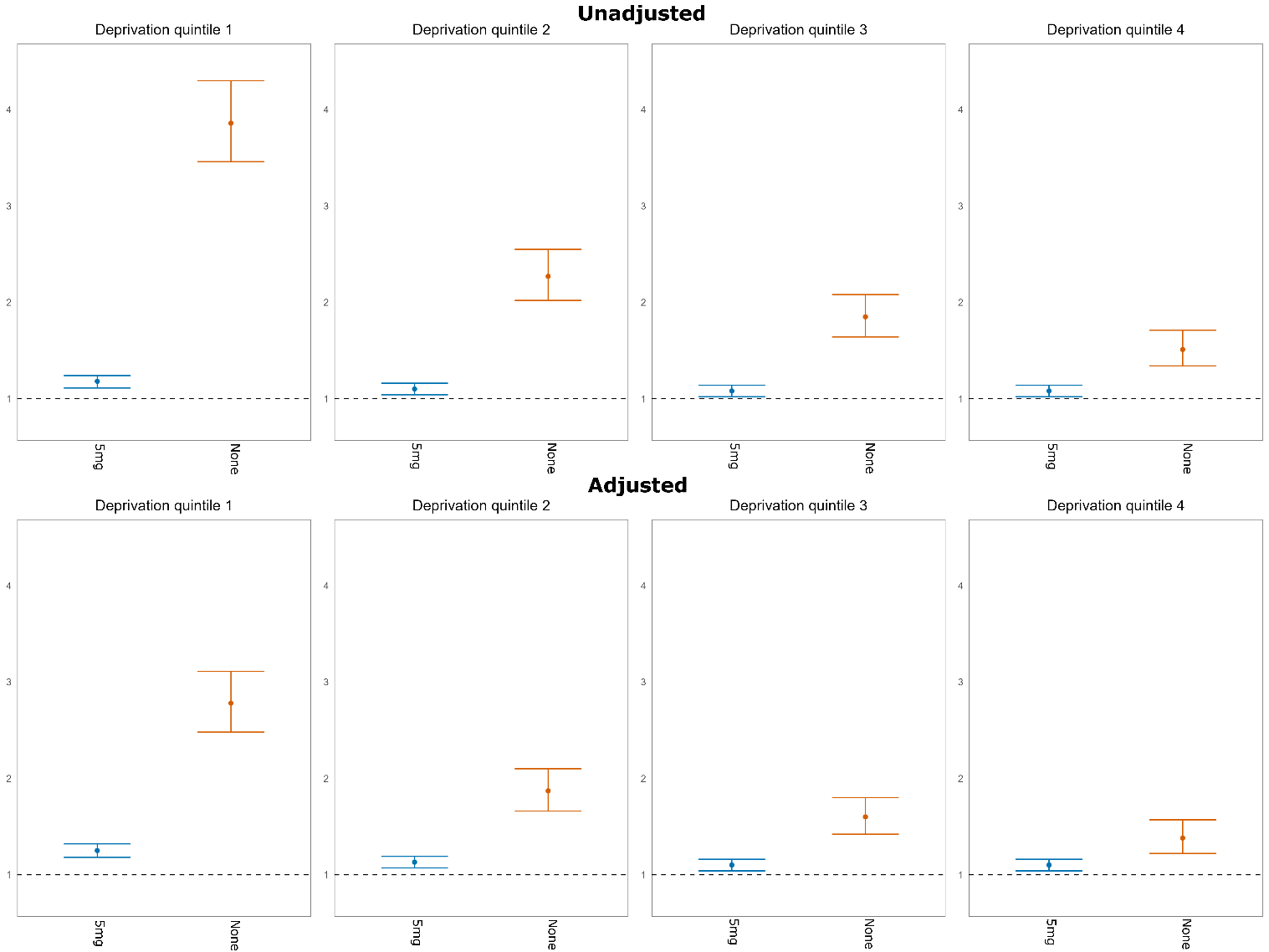


Figure 2. Deprivation vs folic acid dosage (reference: 400mcg): OR (95%CI)


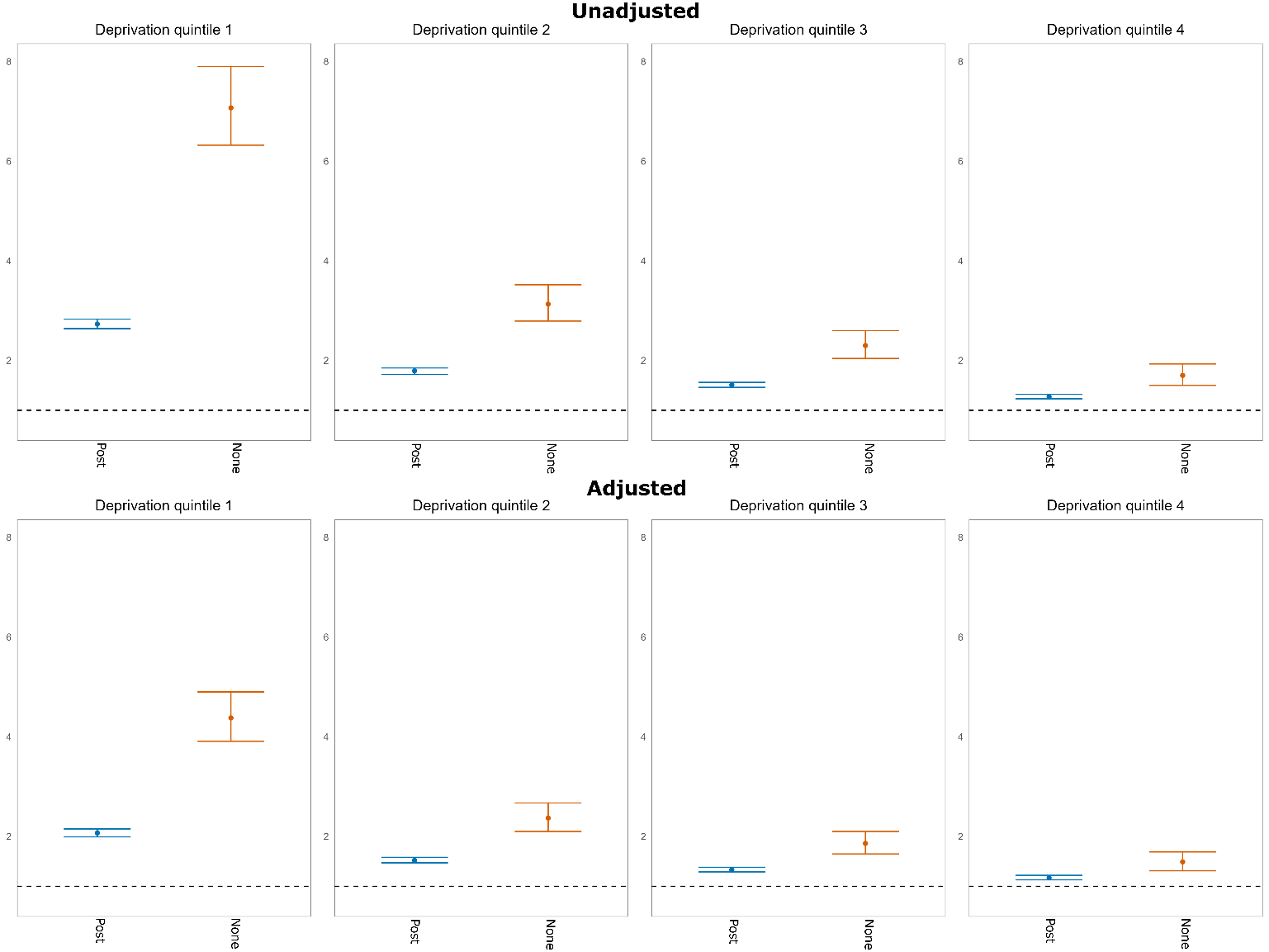


Figure 3. Deprivation vs folic acid timing (reference: preconception): OR (95%CI)
